# Nanoparticles from renewable polymers

**DOI:** 10.3389/fchem.2014.00049

**Published:** 2014-07-18

**Authors:** Frederik R. Wurm, Clemens K. Weiss

**Affiliations:** ^1^Physical Chemistry of Polymers, Max Planck Institute for Polymer ResearchMainz, Germany; ^2^Life Sciences and Engineering, University of Applied Sciences BingenBingen, Germany

**Keywords:** nanoparticles, polymers, biodegradation, formulation of nanoparticles

## Abstract

The use of polymers from natural resources can bring many benefits for novel polymeric nanoparticle systems. Such polymers have a variety of beneficial properties such as biodegradability and biocompatibility, they are readily available on large scale and at low cost. As the amount of fossil fuels decrease, their application becomes more interesting even if characterization is in many cases more challenging due to structural complexity, either by broad distribution of their molecular weights (polysaccharides, polyesters, lignin) or by complex structure (proteins, lignin). This review summarizes different sources and methods for the preparation of biopolymer-based nanoparticle systems for various applications.

## Introduction

Nanoparticles have become omnipresent in science but also have found their ways in a plethora of everyday consumer goods. Synthetic inorganic nanoparticles find application from catalysts to sunscreens and are made from a wide variety of materials, ranging from metals or alloys, to semiconductors, oxides or other ceramic materials (Daniel and Astruc, 2004; Astruc et al., 2005; Kamat, 2007; Vallet-Regi et al., 2007; Na et al., 2009; Becker et al., 2010; Muñoz-Espí et al., 2012). In contrast to these materials, polymeric nanoparticles are designed synthetically for manifold applications. Typically, common vinyl monomer-based polymers, such as polystyrene, polyalkyl(meth)acrylates, few polyesters and polyurethanes are used. Most of them are of synthetic origin. Functionality is added to the particles by chemical modification of the surface. In addition to the general discussion about sustainability (Musto, 2013), and the drive to use “green” processes or raw materials, the naturally occurring polymers or monomers-derived from natural resources- offer great potential for broadening the scope of materials for the preparation of polymeric nanoparticles. In this review, we will briefly introduce polymers and monomers, which are of natural origin or derived from renewable sources, and present some of their unique properties. Subsequently, suitable formulation techniques are discussed and we conclude with recent examples and an outlook to potential future applications.

We will only discuss and present *nano*particle systems and, although there are several examples, not refer to *micro*particles, including microgels.

## Polymers and monomers from natural resources

Polymers from natural resources accompanied mankind throughout history. There is archeological evidence for the use of flax fibers, consisting of cellulose, other polysaccharides, and lignin, already 30,000 years ago (Kvavadze et al., 2009). Although animal derived materials had found their use before, evidence for the use of wool, fur, silk or leather, which are animal derived, protein (keratin, collagen, silk protein) materials, is dating back almost 7000 years (Good et al., 2009). Ancient Mesoamerican people used natural rubber (polyisoprene) in its liquid or colloidal form as medicines, and in its solid form for creating decorative items, coatings, and solid balls to be used in ritual ball games (Hosler, 1999). Widespread application for rubber, however, was enabled by the invention of vulcanization of natural rubber by Goodyear in the nineteenth century. Although polymers from renewable resources have been used for millennia, the recent years witnessed enormous efforts to use plants, animals or microbes as sources for polymers or monomers as alternatives to fossil oil-derived systems, especially in pursuit to match physical, chemical and mechanical properties of synthetic polymers. Extensive literature with excellent reviews presenting details on the different materials have been published recently (Gandini and Belgacem, 1997; Lora and Glasser, 2002; Luengo et al., 2003; Yu et al., 2006; Gandini, 2008, 2011; Kim et al., 2008; Sharma and Kundu, 2008; Vemula and John, 2008; Calvo-Flores and Dobado, 2010; Madhavan Nampoothiri et al., 2010; Mutlu and Meier, 2010; Raquez et al., 2010, 2013; Biermann et al., 2011; Montero de Espinosa and Meier, 2011; Fernandes et al., 2013; Lligadas et al., 2013; Mosiewicki and Aranguren, 2013; Auvergne et al., 2014; Miao et al., 2014). Here, we present a brief overview of some prominent types of polymers and monomers, highlighting their properties and their potential benefits for potential applications of nanoparticulate materials.

### Polysaccharides

**Figure 1:**
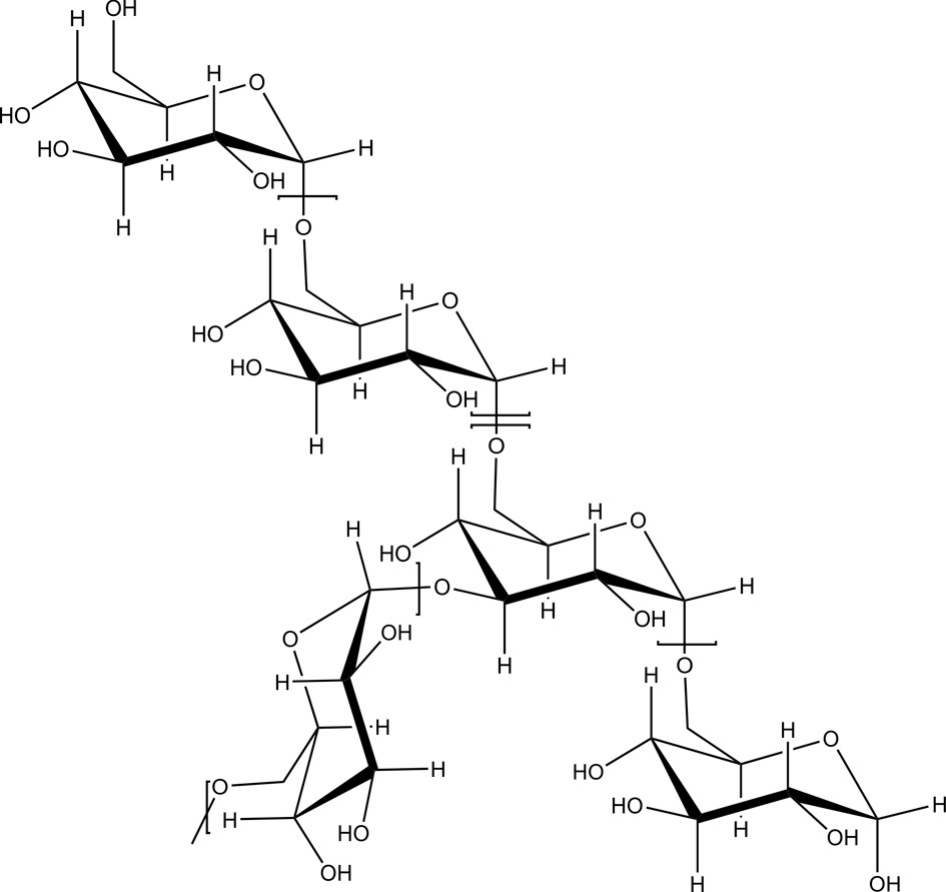
**Dextran as an example of a branched polysaccharide with multiple hydroxyl groups as functional moieties**.

Natural polysaccharides are ideal materials for the preparation of nanoparticles for drug or protein delivery due to many advantages over synthetic polymers such as natural abundance, generally low cost, ease of manipulation, facile derivatization due to the presence of several nucleophilic groups and, in most cases, biocompatibility. Polysaccharides have a high number of functional groups, a wide range of molecular weights with varying chemical and structural compositions, which contribute to their diversity in structure and in physical property. Due to the presence of various derivable groups on the molecular chains, polysaccharides can be easily modified chemically and biochemically, resulting in many kinds of polysaccharide derivatives. As these biomaterials occur naturally, they are typically very stable, hydrophilic, non-toxic and biodegradable. Particularly, most of natural polysaccharides have hydrophilic groups such as hydroxyl (Figure 1), carboxyl and amino groups, which could form non-covalent bonds with biological tissues (mainly epithelia and mucous membranes).

### Polyesters

**Figure 2:**
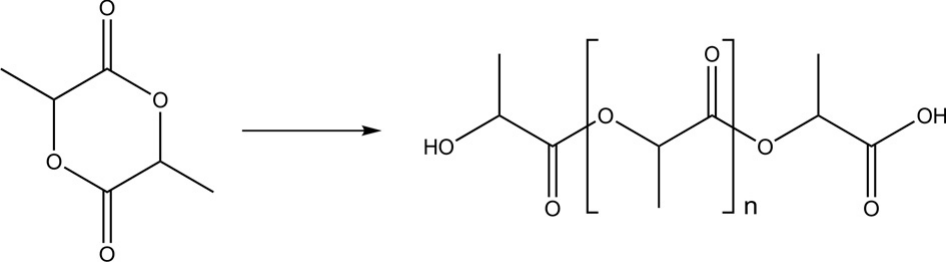
**Schematic synthesis of poly(lactic acid) from lactide**.

**Figure 3:**
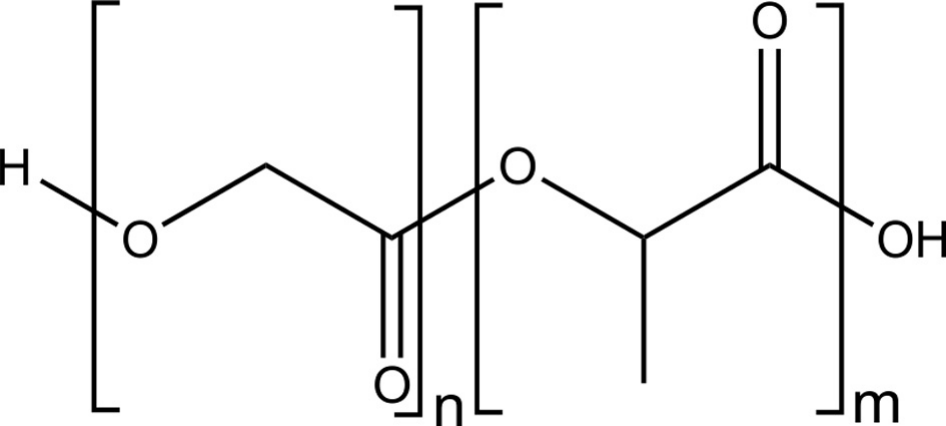
**Structure of poly(lactic acid-*co*-glycolic acid)**.

Polyesters such as polylactide (PLA) (Figure 2) or copolymers of poly(lactide-*co*-glycolide) (Figure 3) are biocompatible and biodegradable materials that are already used as commodity plastics (Gupta and Kumar, 2007; Madhavan Nampoothiri et al., 2010; Kfoury et al., 2013; Raquez et al., 2013) and also widely used in biomedical applications such as drug delivery or in tissue engineering.

This is mainly due to their safe degradation profile and their commercial availability. They have been approved by the United States Food and Drug Administration (FDA) and European Medicine Agency (EMA) for diverse biomedical applications (drug delivery devices, sutures, and implants). As polylactide is a readily available thermoplastic, biodegradable polyester based on renewable resources, it is a promising material for present and future applications, which substitute fossil materials. The monomer lactic acid (or lactide, the cyclic dilactone) is chiral, having two possible enantiomers (D and L), which can be polymerized enantiomerically pure or in their racemic form: poly(L-lactide) (PLLA) or poly(D-lactide) (PDLA), and the racemic mixture, i.e., poly(D,L-lactide) (PDLLA). Polylactides with an L-content above 90% are crystalline materials, while PDLLA is an amorphous polymer due to the random orientation of the pendant methyl side chain. For applications, semicrystalline PLA is often preferred over the amorphous materials especially when higher mechanical stability is necessary (tensile strength ca. 50–70 MPa). PLA has a glass transition temperature (*T*_g_) between 50 and 65°C and a melting temperature (*T*_m_) of ca. 175–180°C depending on the ratio of D:L in the monomer mixture. Copolymers with glycolide are also widely applied due to the varied mechanical and degradation profile. Besides the broad application several factors may limit their use, such as high crystallinity resulting in (too) slow release, or acidic degradation products, i.e., carboxylic acids, which hamper cell growth in some cases.

Other polyesters, such as poly(hydroxy butyrate), which is produced by microbes, have so far not found many applications in nanotechnology.

### Polypeptides/proteins

Proteins/peptides as building blocks for nanoparticles are obvious due to their degradability, usually non-toxicity and easy availability. Proteins are polymers from amino acids, produced by living organisms. They constitute tissues, have regulatory and transport properties, and catalyze metabolic reactions. So far, gelatin (a degradation product of collagen) and albumin have been used for the preparation of nanoparticles. The first commercialized protein nanoparticle-based product was albumin-bound paclitaxel (nab™-paclitaxel; Abraxane®) with a mean diameter of ca. 130 nm. It was approved by the FDA in 2005 for the treatment of breast cancer in patients who fail combination chemotherapy for metastases, or relapse within 6 months of adjuvant chemotherapy (Hawkins et al., 2008). Gelatin nanoparticles were proposed for drug delivery and, in combination with mineralized calcium phosphate, as bone substitute or bone-regrowth substrate (Balthasar et al., 2005; Kommareddy and Amiji, 2007; Ethirajan et al., 2008a,b; Gan et al., 2012; Khan and Schneider, 2013; Xu et al., 2014).

### Lignin

**Figure 4:**
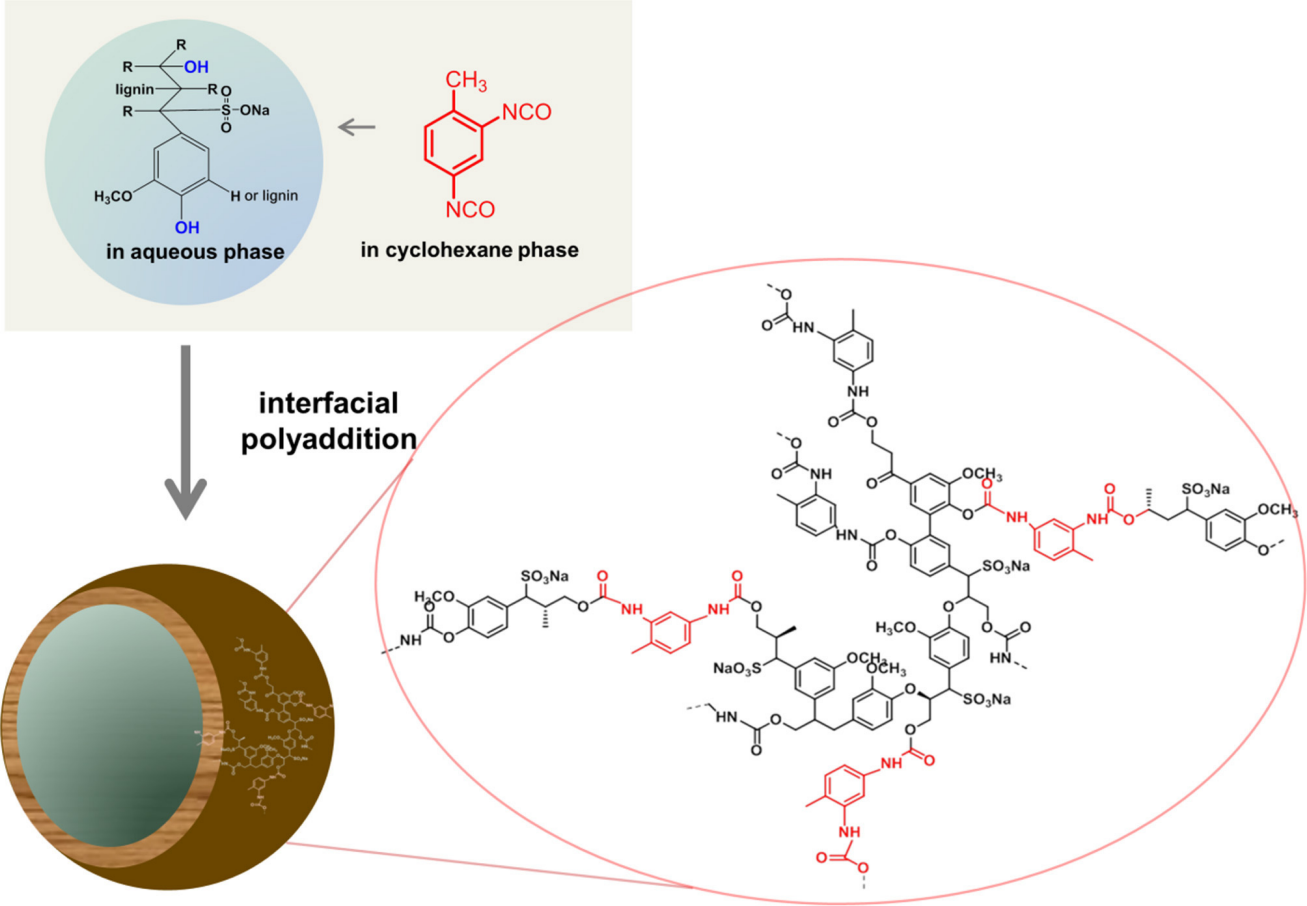
**Schematic representation for the preparation of lignin nanocapsules (image taken from Yiamsawas et al., 2014 - Published by The Royal Society of Chemistry)**.

Lignin is one of the most abundant renewable biopolymers. It is extracted from plants and represents approximately 15–30% of their total biomass besides cellulose as the major component. In spite of the huge amounts of lignin, which are readily available, lignin is normally considered as a non-preferred byproduct from the paper industry for example with over 30 mio t/a (Hatakeyama and Hatakeyama, 2010). A potential reason for considering lignin as a waste product is probably the very limited solubility of the native material and the complexity of the lignin structure with very broad molecular weight distributions and a random microstructure. Thus only 2% of lignin (mainly lignosulfonates) are applied in agricultural uses or other industries as binders, e.g., for animal feed pellets, bricks, ceramics, or road dust, dispersants for oil well drilling products, etc. (Lora and Glasser, 2002; Calvo-Flores and Dobado, 2010). Lignin is a highly branched polyphenolic polyether, with the main structural elements being three “monomeric units,” i.e., 4-hydroxyphenyl, guaiacyl, and syringyl derivatives, which are connected via aromatic and aliphatic ether bonds that build up a hyperbranched, i.e., irregularly branched polymer (Calvo-Flores and Dobado, 2010). Lignin is highly functional and carries both phenolic and aliphatic hydroxyl groups that can be used for further modification or polymerization. Several publications deal with the use of lignin in materials science, however only very few example are present that use lignin in nanoparticle formulations. Recently, lignin was doped with multi-walled carbon nanotubes and used as a macromonomer in a step-growth polymerization with toluene-2,4-diisocyanate (TDI) terminated poly(propylene glycol) for chemical sensoring applications (Faria et al., 2012). In addition, the hydroxyl groups of lignin films were modified with PNIPAM through ATRP under aqueous conditions to prepare ion-responsive nanofibers (Gao et al., 2012). We recently developed a straightforward strategy to generate hollow nanocapsules by interfacial polyaddition of lignin with TDI in an inverse miniemulsion. Lignin derivatives were dissolved in water and dispersed in organic solvent. Upon addition of TDI a polyaddition reaction with lignins' hydroxyl groups occurred at the droplet surface (Figure 4). By this approach hydrophilic substances can be incorporated into biodegradable lignin nanocontainers (Yiamsawas et al., 2014).

### Biogenic monomers

#### Amino acids

Amino acids are ubiquitous in nature as they are the monomeric units of proteins. Although recent studies describe the presence and importance of α-D-amino acids in proteins, the predominant natural form is the L-enantiomer. In addition to the proteinogenic amino acids, chemical functionality can easily be synthetically introduced into amino acids offering an extremely wide variety of possible applications. Amino acids are typically commercially available with fluorenylmethoxycarbonyl (Fmoc) or *t*-butoxycarbonyl (Boc) protected amino groups. In these forms they are suitable for controlled solid phase peptide synthesis. Peptides are sequentially constructed on a resin, offering structural and chemical control over the generated peptides. The obtained peptides can be used for pharmaceutical purposes, but also as structural motif in peptide polymer conjugates, as intermolecular and intramolecular interactions can be tuned by the peptide sequence. This determines the peptide structure and can eventually control the self-assembly behavior of peptide-polymer conjugates (Gauthier and Klok, 2008; Lutz and Börner, 2008; Krishna and Kiick, 2010). Introduced in nanoparticulate systems, they were used as cleavage site for release of encapsulated materials, but also for the recognition and optical detection of enzymes (Maier et al., 2011; Andrieu et al., 2012; Fuchs et al., 2013).

#### From polysaccharides

Polysaccharides can also serve as raw materials for the generation of fine chemicals, such as monomers for polymerization. Examples are bio-ethanol, which can be converted to ethylene glycol for polyaddition (PET) (Jang et al., 2012), or furan-based systems (Gandini and Belgacem, 1997; Gandini, 2008, 2011; Pranger and Tannenbaum, 2008; Gandini et al., 2009).

#### From triglycerides

Phospholipid vesicles, i.e., liposomes, have found increasing attention as nano-drug carriers since several decades. Additionally, oils and fatty acids derived from plants are currently discussed as versatile sources for the preparation of different products, including polymers and polymeric nanoparticles (Biermann et al., 2011). Since the beginning of the 1990s solid lipid nanoparticles (SLN) have been discussed as alternatives to synthetic polymer particles, due to their good biocompatibility, large-scale production and cost-effective formulation. The use of lipid pellets was already known for oral drug delivery, e.g., Mucosolvan(R) retard capsules, which was followed by lipid microparticles made by spray congealing (Eldem et al., 1991) and nanoparticles from microemulsion (Schwarz et al., 1994). A typical argument as an advantage of SLNs over polymeric nanoparticles is that they can be produced by high pressure homogenization identical to conventional oil in water emulsions (Müller et al., 2000), a process, which can be easily conducted industrially on large scale. Loading capacities are reported for several drugs and vary strongly with drug administered from a few percent for retinol (Westesen et al., 1997) for example up to 50% for ubidecarenone (i.e., Coenzym Q10) which is mainly related to the solubility of the drug in the lipid (Jenning et al., 2000). This loading capacity is also reflected in the drug release, which is often found as a burst release. Some examples present a release up to several weeks (5–7 weeks) by variation of the lipid matrix, surfactant concentration and production parameters (such as temperature) (Zur Mühlen and Mehnert, 1998).

Fatty acids can be derived from vegetable oils by hydrolysis (Mutlu and Meier, 2010; De Lima et al., 2013). They have been intensively studied for the use as building blocks in polymer science and derivatized to a variety of functional monomers. Recently, metathesis polymerization and used to prepare nanoparticles by miniemulsion. A strong dependence on the nature of the catalyst and surfactant was found; however the removal of Ru-based catalysts may be challenging in such systems (Cardoso et al., 2014). Also the encapsulation of several vegetable oils by emulsion techniques has been investigated (Cardoso et al., 2013). Jojoba and andiroba oil were encapsulated into polymeric nanostructures by miniemulsion polymerization. The effects of the addition of different hydrophilic monomers such as acrylic or methacrylic acid with the oils being co-stabilizers were evaluated. The formation of hollow (oil-filled) nanocapsules was investigated. Fatty acids were also used to prepare nanoparticles from protein-fatty acid ionic, i.e., salt, complexes (Yoo et al., 2001). Lysozyme was modified with hydrophobic fatty acid salt, i.e., sodium oleate, via ionic binding between the anionic carboxylate and the basic amino groups in lysozyme. The lysozyme-oleate complex dissolved in an organic solvent exhibited much higher conformational stability at elevated temperature than free lysozyme in the same solvent. The complex was formulated into biodegradable nanoparticles by a spontaneous emulsion and solvent diffusion method. The resultant formulation showed near 100% encapsulation efficiency of lysozyme within nanoparticles with <100 nm in diameter with a narrow size distribution. These biodegradable nanoparticles showed efficient encapsulation of proteins which are potentially useful for oral protein delivery including mucosal vaccination.

### Others

Glycolipids are complex materials that can be produced by fermentation by yeast (such as *Candida bombicola*) from a mixture of carbohydrates and lipids. One class, the so called sophorolipids can be produced by fermentation of *C. bombicola* on glucose/oleic acid mixtures resulting in a mixture of several components including lactonic and acidic forms. Natural sophorolipids and selected derivatives are currently discussed for surfactants, emulsifiers, and therapeutic agents (antibacterial, antifungal, septic shock, anticancer, etc.) (Guilmanov et al., 2002; Zini et al., 2008). They have been polymerized via various polymerization techniques such as ring-opening metathesis polymerization and represents a promising class of materials for future development of drug-delivery vehicles for example.

Other plant derived chemicals as, e.g., terpenes have also been proposed for application in polymer science (Wilbon et al., 2013).

## Preparation techniques

Nanoparticles can be formulated by basically three ways: (1) the monomer is polymerized during the preparation process to eventually form a nanoparticulate system, (2) an insoluble polymer is subjected to a physical process resulting in nanoparticles, (3) a soluble polymer is cross-linked in a suitable way. (1) Comprises classic or emulsifier free emulsion polymerization, miniemulsion polymerization and microemulsion polymerization, dispersion polymerization usually leads to microspheres. (2) Comprises (mini)emulsion solvent evaporation, emulsion solvent shifting or nanoprecipitation (Ouzo-effect). (3) Describes the formation of nanogels or crosslinked polymeric particles.

### Nanoprecipitation/Ouzo-effect

**Figure 5:**
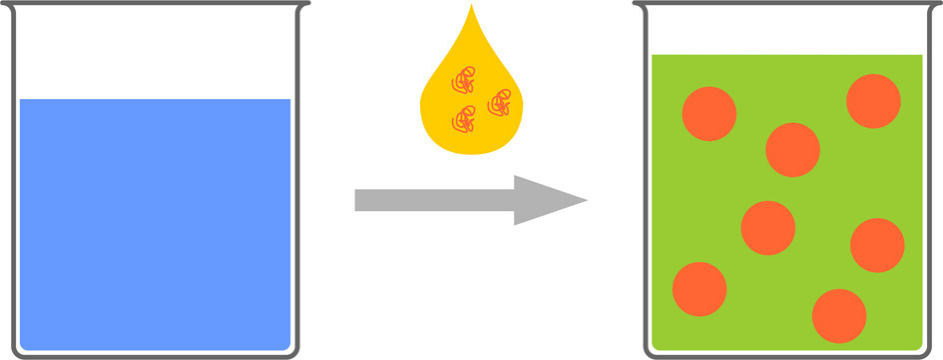
**Schematic representation of the polymeric Ouzo-effect**. A polymeric solution is added to water. Nanophaseseparation leads to polymeric nanoparticles.

Several terms can be found in the literature for the process, where a solution of an organic compound in a water miscible organic solvent is mixed with an excess of water and nanoparticles are generated by a phase separation process (Figure 5). Most widely used are the terms: nanoprecipitation, solvent shifting, or “Ouzo-effect” (Thioune et al., 1997; Vitale and Katz, 2003; Ganachaud and Katz, 2005; Hornig et al., 2009; Schubert et al., 2011; Aschenbrenner et al., 2013).

This phenomenon is well known from alcoholic beverages containing the oil of the anise seed, such as Ouzo or Pastis. These drinks are, chemically spoken, a solution of anethol (and some other components) in water/ethanol. When additional water is added to this solution, the solubility limit of anethol is passed and phase separation occurs. Interestingly, no macroscopic phases are initially generated. In contrast, submicron droplets with high stability are generated, resulting in the cloudy appearance of the drink after water addition. In other words, an aqueous dispersion of nanoparticles/droplets is generated without the necessity of additional energy input, which makes the process mild and cheap.

This process also finds application in the formulation of pigments (Horn and Rieger, 2001; Van Keuren et al., 2003, 2008; Chen et al., 2009), and has massively been used for the preparation of polymeric nanoparticles, especially polyester based, for pharmaceutical applications (Brick et al., 2003; Liebert et al., 2005; Miller, 2006; Hornig et al., 2009; Beck-Broichsitter et al., 2010; Mora-Huertas et al., 2010, 2011). Especially for the encapsulation of sensitive drugs, the absence of energy input is a great benefit.

Although a variety of solvent/polymer combinations have been evaluated, the mechanism of formation and stability is not completely elucidated. Despite the wide applicability and the ease of the system, the major drawback may be the low (around 1 wt%) solids contents which can be obtained (Brick et al., 2003; Vitale and Katz, 2003; Ganachaud and Katz, 2005; Aubry et al., 2009).

### Emulsion based processes

Classic emulsion polymerization finds major application in the industrial production of (meth)acrylic and vinyl polymers via radical polymerization (Harkins, 1947). As the monomer has to diffuse through an aqueous phase to reach the place of polymerization, very hydrophobic monomers are difficult to process. Additionally, other polyreactions besides the free radical polymerization are difficult to handle. Preformed polymers cannot be formulated to nanoparticles using the emulsion polymerization technique. In contrast to the above mentioned phase separation stable preformed emulsions can be used as templates or enclosed reaction vessels of the dimensions of only few microns or several hundreds of nanometers for polyreactions or phase separation processes.

**Figure 6:**
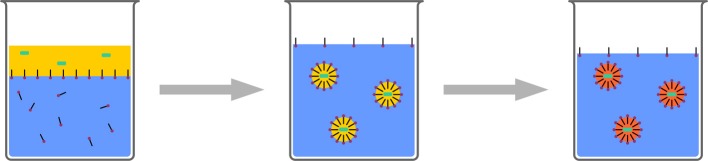
**Schematic representation of miniemulsion polymerization**. Stable droplets in the range of few hundreds of nanometers are generated by the addition of a costabilizer (light green) and homogenization by ultrasound. The monomer droplets act as nanoreactors.

The stability of droplets in emulsions is governed by two processes: First, droplets fuse by collision and coalescence, and second, droplets degrade by diffusion (Ostwald Ripening). Coalescence can be controlled by the addition of surfactants to the emulsions. These create an electrostatic or steric barrier around the droplets, creating a repulsive force between the droplets. Ostwald Ripening is caused by the imbalance of Laplace pressures between droplets of different sizes. Higher curvature leads to higher Laplace pressure, forcing the contents of smaller droplets to diffuse into larger droplets. This can be counteracted by an as-narrow-as-possible droplet size distribution, and by the addition of a so-called costabilizer to the droplets. The presence of this costabilizer, which is a component with a very low solubitiy in the continuous phase, creates an osmotic pressure, opposite to the Laplace pressure. Consequently, diffusion is limited. Such systems are typically referred to as miniemulsions. The droplets stabilized in this manner can be used for various polyreactions and physical processes for nanoparticle formulation (Figure 6).

The stability of the droplets in miniemulsions can be used in several ways for the formation of nanoparticluate systems. Depending on the formulation technique, nanoparticles, nanocapsules, or nanogels can be created. Detailed reviews about the mechanism and the preparative possibilities have recently been published (Landfester et al., 1999a,b; Landfester, 2000, 2009; Asua, 2002, 2004; Schork et al., 2005; Crespy and Landfester, 2010; Landfester and Weiss, 2010; Weiss and Landfester, 2010).

### Emulsion/solvent evaporation

**Figure 7:**
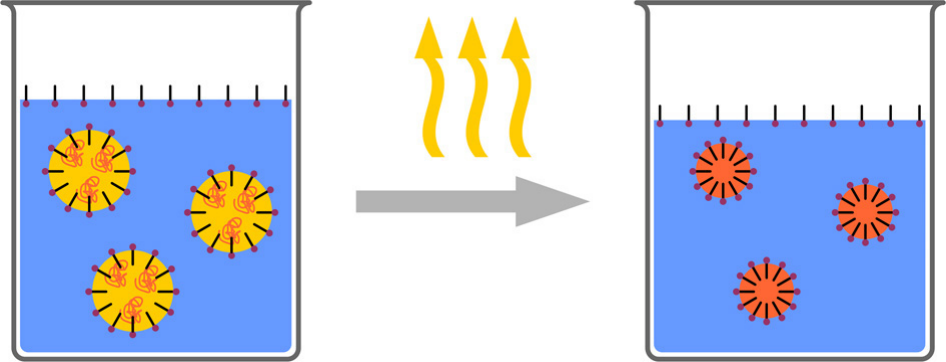
**Schematic representation of the solvent evaporation process**. A polymer solution is formulated as a surfactant stabilized emulsion. Subsequently the solvent is evaporated resulting in polymeric nanoparticles.

In contrast to the above mentioned technique of nanoprecipitation, where phase separation for the formation of nanoparticles is induced in the bulk of the solution, phase separation can also be induced in droplets (Figure 7). Such is typically achieved by dispersing a polymer solution in a surfactant solution (typically aqueous) to form stable droplets. Either the polymer is precipitated by a water soluble organic solvent, diffusing from the droplet to the aqueous phase or by evaporating the solvent from the droplets (Mora-Huertas et al., 2011; Staff et al., 2013). Both lead to nucleation of the solid polymer, which starts at the droplet/solvent interface (Anton et al., 2008). As the solvent from the droplets is removed through the continuous phase, the miscibility of the solvents is crucial for evaporation and consequently for the (nano)particle formation. Solvent, temperature, pressure, and stirring speed are parameters which need to be controlled. Eventually, when the entire solvent has been removed, solid polymeric particles-stabilized by the surfactant which was initially added for stabilizing the droplets-form a stable dispersion.

The process is conceptually easy and is principally suitable for all polymers, for which a good solvent can be found. Furthermore, the process is quite fast (few hours) and, in contrast to polymerization techniques, no residual monomer or initiator can be found in the dispersion. Drawbacks are mainly the broad particle size distribution, the low solids contents (few %) and residual surfactants. Concentrating and cleaning may overcome the latter both issues; they are however, time consuming discontinuous steps, which may prevent large scale application.

The use of not only one dissolved component in the dispersed phase reveals another exciting topic in this area. Depending of the wetting properties (interfacial energies) (Torza and Mason, 1970) of the dissolved components, the phase separation can be directed in such a way, that complex particle morphologies can be generated. Given enough time for complete phase separation, the morphology adopted presents a minimum of the Gibbs Free Enthalpy. These ideal equilibrium morphologies are core-shell, partially engulfed (snowman-like), and separated. In real systems, several factors such as the viscosity of the solution, the temperature, and the amount of surfactant, which influences interfacial energies, determine the final morphology of the particle. This can be one of the ideal morphologies, capsules if one component is liquid, but also multi-compartment structures and other kinetically controlled structures. A review about the features, the mechanism and the scope of the emulsion solvent evaporation process was recently published (Staff et al., 2013).

### Emulsion/polyreactions

**Figure 8:**
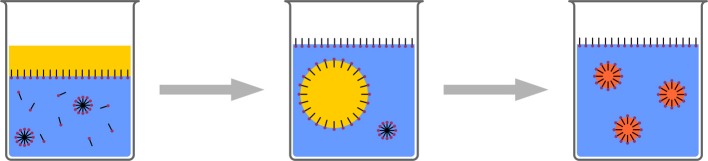
**Schematic representation of classic emulsion polymerization**.

In the classic emulsion polymerization process, acrylic or vinylic monomers are dispersed in an aqueous surfactant solution by mechanical stirring. A water-soluble initiator for free radical polymerization is also added to the system. The surfactant concentration is higher than the critical micelle concentration (cmc). Polymerization is initiated in the aqueous phase, leading to water-insoluble oligomers, entering micelles containing the monomer. Here, polymerization is maintained, as further monomer is diffusing through the aqueous phase to the locus of polymerization (Figure 8). A modification is the so called soap-free emulsion polymerization (Goodwin et al., 1974). Here, the monomer is dispersed in an aqueous solution of initiator, which is typically a peroxodisulfate. Thus, polymerization is also initiated in the aqueous phase. The sulfate terminated oligomers act as surfactants, generating micelles, which then act as locus for polymerization. Eventually, polymeric particles of few hundreds of nanometers or smaller are generated. To initiate polymerization and sustain diffusion in a sufficient way, a certain water solubility of the monomers is required for both techniques. Although there is a report about polycondenstation in emulsion (Jönsson et al., 2013), leading to polyester particles, classic emulsion polymerization was mainly found to be suitable for free radical polymerization of sufficiently water-soluble acrylic or vinylic monomers.

**Figure 9:**
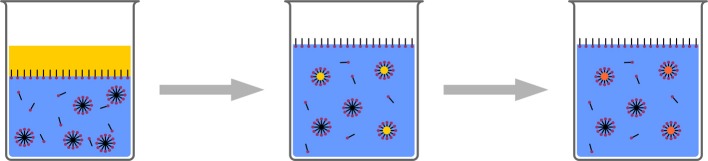
**Schematic representation of microemulsion polymerization**. Radical polymerization in very highly concentrated surfactant/co-surfactant solution leads to ultrasmall polymeric nanoparticles.

In addition to microemulsion polymerization (Pavel, 2004; McClements, 2012) where aqueous solutions with very high surfactant concentration and additional co-surfactant (medium chain alcohol) is used (Figure 9), miniemulsions have shown to be a versatile platform for a variety of polyreactions (Crespy and Landfester, 2010, 2011).

The above described features of miniemulsions allow among others conducting free radical polymerization of “normal” monomers and very hydrophobic monomers, even fluorinated monomers (Landfester et al., 2002), controlled radical polymerization (Bombalski et al., 2007; Esteves et al., 2007; Oh et al., 2007; Siegwart et al., 2009), anionic (Limouzin et al., 2003; Weiss et al., 2007a,b) and cationic polymerization (Cauvin and Ganachaud, 2004), catalytic polymerization (Cardoso et al., 2014; Malzahn et al., 2014b), as well as polyaddition and polycondesation reactions (Landfester et al., 2000; Tiarks et al., 2001; Barrère and Landfester, 2003). Each of these reactions can be used for the preparation of aqueous dispersions of polymeric nanoparticles. The major benefit of emulsion polymerization techniques is clearly the high achievable solid content, and the fact that this technique is already established in industry.

### Emulsion/interfacial reactions/crosslinking

**Figure 10:**
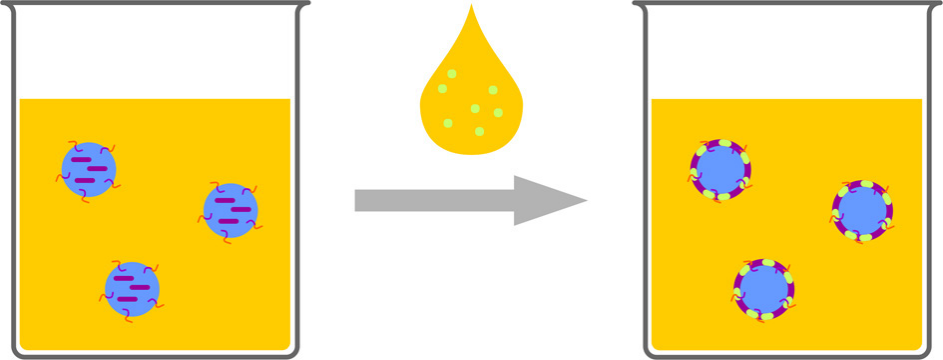
**Schematic representation of interfacial reactions in inverse emulsion**. One monomer (purple) is provided in the aqueous droplets, the other one (light green) is added, dissolved in solvent. The reaction leads to a polymeric shell enclosing the water droplets.

Polymerization can be conducted at interfaces-a very famous example is the “nylon rope trick.” Two immiscible solutions are overlaid, one containing a diamine the other one a diacid chloride. The product of the polycondensation, which can only proceed at the interface, i.e., a Schotten–Baumann reaction, can be drawn out of the biphasic system to produce a nylon thread. Translating such a system to the nanoscale can be used for creating a polymeric shell or membrane at the interface of a droplet in an emulsion. Aiming at nanocapsules with aqueous core, this classic reaction is not suitable, as the diacid chloride reacts to the unreactive diacid, upon contact with water which is a “dead end” and cannot participate in polymerization. Besides a very special monomer pair (hydrophilic vinyl ether and hydrophobic maleates) (Scott et al., 2005; Wu et al., 2006) for strictly alternating radical polymerization and the metathesis polymerization of acrylated dextran and unsaturated organophosphates (Malzahn et al., 2014b), mainly polyaddition reactions of a polyol or a polyamine with diisocyanates to generate polyurethane or polyurea are reported (Crespy et al., 2006). An aqueous solution of the water soluble component, such as low molecular compounds (Paiphansiri et al., 2009), polysaccharides (Crespy et al., 2006; Baier et al., 2012, 2013), or peptides (Andrieu et al., 2012), is dispersed in a solution of a surfactant in an inert solvent, typically a hydrocarbon, such as cyclohexane or isooctane. The other reactive component, e.g., toluene diisocyanate is added to the mixture, diffuses to the droplets and reacts at the interface with the alcohol or amine comonomer, or with water (Figure 10). The reaction with water generates a diamine, which can also act as comonomer and, in contrast to the diacid chloride, is not lost for polymer generation.

If each monomer is insoluble in the other solvent, the reaction proceeds only at the interface. If the organo-soluble monomer shows certain solubility in the dispersed phase, polymerization can also proceed in the droplet. Once the molecular weight is high enough the polymer can become insoluble and precipitate at the interface. Thus, changing the quality of the solvent can be used for tuning the morphology from particle to capsule (Andrieu et al., 2012).

Creating droplets of a preformed functional polymer and adding a crosslinker can be used for the preparation of highly crosslinked organic nanoparticles or nano-scaled hydrogels (Maier et al., 2011; Klinger et al., 2012; Malzahn et al., 2014a).

## Applications

The benefits of polymers from renewable resources are at the moment probably most prominent in applications in biomedicine. As outlined above, one of the major benefits of such systems is biocompatibility and the specific biochemical (enzymatic) or chemical (hydrolytic) degradation, which are of great importance, when such a system is proposed for application in living organisms. In addition to the use of proteins, especially gelatin, for the formulation of nanoparticles, the most of the relevant systems found in literature are based on polysaccharides and polyesters, especially poly(lactic acid). Indeed, besides commercially available polystyrene beads, nanoparticles made from poly(lactic acid) or derivatives are the most used polymeric nanoparticulate systems in biomedicine. Only part of the huge number of publications can be accounted for in this section. We hope to give an idea about the possibilities and refer the reader to comprehensive reviews about nanoparticles for biomedical applications.

### Polysaccharides

As polysaccharides are typically hydrophilic and water soluble, formulating these polymers as nanoparticles in aqueous dispersion requires either hydrophobization prior to formulation, or crosslinking the polymer to form nanoscaled hydrogels. When the crosslinking reaction is conducted at the interface of a droplet, nanocapsules with liquid core can be generated (see above). Typically, the crosslinking with hydrophobic agents leads to impermeable shells. If the crosslinking reaction is conducted in a droplet containing the polysaccharide, nanogels are created. Rather recent reviews by Janes et al. (2001), Prabaharan et al. (Prabaharan and Mano, 2005), and Liu et al. (2008) describe many polysaccharide nanoparticle carriers in detail with respect to synthesis and applications. Herein, the fundamental and the most recent reports on nanoparticle preparation based on polysaccharides are addressed. Hydroxy ethyl starch (HES) based on natural starch, which is modified with ethylene glycol units, has proven to exhibit a stealth effect similar to PEG (see below, polyesters). The reduced protein adsorption of HES was already proven by several pharmaceuticals that are modified with it (Noga et al., 2012; Liebner et al., 2014). As a typical cross-linker for polysaccharides in early reports glutaraldehyde was frequently applied, e.g., for chitosan-based nanoparticles (Zhi et al., 2005), however the toxicity of glutaraldehyde limits its application in drug delivery as residues are difficult to remove from the crosslinked particles but may leak out during application. A more promising approach was established by the use of water-soluble carbodiimides, condensing the polysaccharide with natural di- or tricarboxylic acids, such as succinic acid, malic acid, tartaric acid, and citric acid. This condensation reaction was very efficient for chitosan particles as the reaction product is an amide. Such nanoparticles were stable in aqueous media at low pH, neutral, and mild alkaline conditions. In the swollen state, the average size of the particles was in the range of 270–370 nm depending on the pH (Bodnar et al., 2005). A convenient way to prepare nanocapsule structures, i.e., an aqueous liquid core, surrounded by a polysaccharide based shell, uses the OH-groups of polysaccharides for crosslinking with di-isocyanates at the interface of an aqueous droplet in an inverse emulsion. With the procedure described above, stable or degradable capsules were created. Dextran, hydroxyethyl starch, or hyaluronic acid was used and typically crosslinked with toluenediisocyanate. Baier et al. recently reported hyaluronic acid based nanocapsules containing an antibacerial agent. In the presence of bacteria (*Staphylococcus aureus* and *Escherichia coli*), which secrete hyaluronidase, an enzyme degrading hyaluronic acid, the capsules were opened and the bactericidal agent released, resulting in an inhibition in bacterial growth (Baier et al., 2013). The hydroxyl groups of polysaccharides can be transformed into many different reactive groups in order to use them for a subsequent polymerization/cross-linking step in a dispersed medium. Acrylation or Methacrylation are typical ways for the introduction of polymerizable groups, which additionally alter the solubility profile of the polysaccharide and allow their dispersion with an organic solvent in water or -if only a few crosslinking units are attached- in an inverse system, i.e., water in oil. We recently prepared hollow nanocapsules based on acrylated dextran which was crosslinked via olefin cross metathesis at the interface of water droplets in an organic continuous phase containing a diolefin phosphate as the comonomer. The olefin-cross metathesis was tailored to the interface of a miniemulsion for the design of novel fully degradable polysaccharide-*co*-polyphosphate nanoparticles. Dextran was modified with acrylate side groups which reacted at the water-oil-interface with hydrophobic diolefin phosphates to generate hollow nanocapsules. The Ru-catalyst remained in the organic continuous phase making a purification to obtain metal-free nanocapsules feasible (Malzahn et al., 2014b). Hydrophobically modified polysaccharides, e.g., by esterification, have been used for the preparation of water dispersible nanoparticles, typically by nanoprecipitation processes (Liebert et al., 2005; Hornig and Heinze, 2007, 2008; Hornig et al., 2008). The possibility for encapsulating small molecules was shown and confirmed by fluorescence correlation spectroscopy (Aschenbrenner et al., 2013).

### Polyesters

As mentioned above polyesters are typically hydrophobic polymers which are degraded hydrolytically and are well tolerated by biological systems. Most of the work found in literature uses poly(lactic acid) “polylactide,” and copolymers with glycolic acid, PEG, or polysaccharides. Nanoparticles from poly(ε-caprolactone) are also reported. Typically, the material is used in its polymeric state. Thus, phase separation techniques for the generation of nanoparticles or nanopcapsules are mostly used. Hydrophobic and hydrophilic fluorophores (Bourges, 2003; Musyanovych et al., 2008), or drugs were encapsulated in pure PLA or in more complex copolymers (Niwa et al., 1993; Jeong et al., 2006a,b; Beck-Broichsitter et al., 2010). Capsules with diclofenac dissolved in an oil core were reported by Guterres et al. (1995) prepared with the nanoprecipitation technique. The preparation techniques also allow the protection of large biomolecules, as plasmid DNA (Perez et al., 2001) and proteins (Pillay et al., 2013; Zaric et al., 2013) as well as the encapsulation of hydrophobized magnetic iron oxides in PLA based nanoparticles. Using the emulsion/solvent evaporation technique, Urban et al. (2009) prepared poly-L-lactic acid (PLLA) nanoparticles of around 100 nm with almost 40 wt% of iron oxide. Encapsulation did not alter the magnetic properties of the iron oxide. Larger particles of 300 nm to 3 μm containing iron oxide were used by Xu et al. (2012) for tracking mesenchymal stem cells with MRI for almost 1 month. Cell functions and properties were not disturbed by the presence of the particles, allowing long term tracking, e.g., of cell migration. Nanoparticles prepared from hydrophobic polyesters are usually readily uptaken by cells via endocytosis (Mailander and Landfester, 2009). If the material is released from the endosome or partly degraded in it, an efficient drug delivery is observed. A typical drawback for all nanoparticles is the adsorption of plasma proteins after the injection in a living organism, resulting in an altered cell uptake or faster degradation of the nanoparticles before reaching the target site (Torchilin and Trubetskoy, 1995; Sahil et al., 1997; Owens and Peppas, 2006). To overcome this problem many particles are modified with poly(ethylene glycol) (PEG), which reduces the amount of adsorbed protein due to the so called “stealth effect,” which increases blood half life time by several orders of magnitude. The so called “PEGylation” is well-known for nanoparticles or molecularly dissolved drugs as the highly hydrated polyether reduces protein adsorption dramatically, (Bazile et al., 1995; Torchilin and Trubetskoy, 1995; Peracchia et al., 1999a,b; Andrieux and Couvreur, 2009) however, PEG is not degraded *in vivo* making a search for degradable alternatives in combination with degradable drug carriers necessary (Liebner et al., 2014) (see polysaccharides). Encapsulated material can either be released by diffusion from the polymeric matrix of the nanoparticles, and seems to depend on several factors as the preparation conditions, and the solubility of the drug in the dispersion medium. The reports range from few minutes to several days (Bourges, 2003; Pillay et al., 2013). The degradation of the polymeric matrix seems to play a minor role, only, as it is quite slow at physiological pH. In fact, diclofenac could be almost completely retained in PDLA for 8 months (Guterres et al., 1995). Control of the degradation properties was achieved by using graft copolymers, consisting of a polysaccharide backbone and grafted PLA (Nouvel et al., 2009) or PLGA chains. Polysaccharide specific enzymes degraded the backbone leading to decomposition of the whole nanostructure, leading to faster release than in the absence of the enzyme (Jeong et al., 2006a,b). Zaric et al. report a delivery system based on a combination of microneedles and antigen loaded PLGA nanoparticles. Microneedles from quickly degrading poly(methylvinylether-*co*-maleic anhydride) are used to deliver PLGA nanoparticles loaded with ovalbumin as model antigen. Encapsulation increases antigen stability in the microneedles and facilitates the targeting and activation of dendritic cells in the skin, opening up a promising route for vaccination (Zaric et al., 2013). Another, so far neglected, class of polyesters found recently more attention, namely polyesters based on phosphoric acid. Similar to DNA or RNA, these materials are connected by main-chain phosphates that are typically derived from phosphorus oxychloride but can also be made from natural occurring sodium phosphate by enzymatic esterification. Several synthesis strategies have been reported ranging from classical polycondensation, via anionic ring-opening polymerization to metathesis polymerization. Moreover, the materials have been investigated in various emulsion and micellar approaches, also in combination with polyesters such as poly lactide or DNA in cation/anion complexes for gene delivery (Mao and Leong, 2005; Zhang et al., 2012; Shen et al., 2013; Steinbach et al., 2013; Alexandrino et al., 2014; Baier et al., 2014).

## Conclusion and outlook

The properties of nanoparticles from renewable resources are currently mainly exploited in biomedical sciences. Specific or unspecific degradation of the polymeric matrix is used for sustained release of encapsulated drugs or as probe for specific environments, e.g., the presence of enzymes or pH regimes. Despite all benefits and promising results, most of these applications, however, are far from being suitable for the industrial scale or a market-ready product. Environmental benignity does not play a major role here, as the prospective quantities are relatively low. This is probably due to the still low costs and established procedures for common vinyl monomers, etc., which are applied in industry today. Furthermore, the structural variety of many natural polymers makes their selective application challenging, broad molecular weight distributions, many different electrophilic and nucleophilic groups-sometimes in the same material- make their chemical modification and further postmodification more difficult compared to fully synthetic polymers. Additionally, the solubility profiles are usually different compared to industrial products [they dissolve in water (proteins, saccharides) or are hardly soluble at all (lignin)] making selective chemistry challenging as all other components must tolerate the aqueous conditions for example.

A prospective large scale application for polymeric nanoparticles from renewable resources may be found as major constituent of paints. For this application, the performance of the polymers has to match these of synthetic polymers and a suitable formulation technique has to be found. For these reasons, monomers derived from plant oils and formulated in conventional, emulsion polymerization based processes are probably most promising, as raw materials are readily available and polymerization techniques are established and allow the generation of dispersions with high solids contents.

### Conflict of interest statement

The authors declare that the research was conducted in the absence of any commercial or financial relationships that could be construed as a potential conflict of interest.
